# Substance Use Disorders and Psychoactive Drug Poisoning in Medically Authorized Cannabis Patients: Longitudinal Cohort Study

**DOI:** 10.1177/07067437211060597

**Published:** 2021-11-20

**Authors:** Arsène Zongo, Cerina Lee, Jihane El-Mourad, Jason R. B. Dyck, Elaine Hyshka, John G. Hanlon, Dean T. Eurich

**Affiliations:** 1 Faculty of Pharmacy, 4440 Université Laval, Quebec City, Canada; 2 Population Health and Optimal Health Practices Research Unit, CHU de Québec - Université Laval Research Centre, Quebec City, Canada; 3 School of Public Health, 3158 University of Alberta, Edmonton, Canada; 4 Cardiovascular Research Centre, Department of Pediatrics, Faculty of Medicine and Dentistry, 3158 University of Alberta, Edmonton, Canada; 5 St. Michael's Hospital Department of Anesthesia, 177410 University of Toronto, Ontario, Canada; 6 Department of Anaesthesiology and Pain Medicine, University of Toronto, Ontario, Canada

**Keywords:** medical cannabis, substance use, poisoning, emergency department, hospitalization

## Abstract

**Objectives:**

Poisoning from psychoactive drugs and substance use disorders (SUD) have been reported among non-medical cannabis users. However, little is known about medical cannabis users and their risk for poisoning and/or development of SUD. This study assessed the risk of emergency department (ED) visits or hospitalization for 1) poisoning by psychoactive drugs and 2) mental/behavioural disorders due to the use of psychoactive drugs and other substances, in medically authorized cannabis patients in Ontario, Canada from 2014–2017.

**Methods:**

A cohort study of adult patients authorized for medical cannabis that were matched to population-based controls. ED visit/hospitalization were assessed with a main diagnostic code for: 1) poisoning by psychoactive drugs; 2) mental and behavioural disorder due to psychoactive drugs or other substance use. Conditional Cox proportional hazards regressions were conducted.

**Results:**

18,653 cannabis patients were matched to 51,243 controls. During a median follow-up of 243 days, the incidence rate for poisoning was 4.71 per 1,000 person-years (95%CI: 3.71–5.99) for cases and 1.73 per 1,000 person-years (95% CI: 1.36–2.19) for controls. The adjusted hazard ratio (aHR) was 2.45 (95%CI: 1.56–3.84). For mental/behavioural disorders, the incident rates were 8.89 (95% CI: 7.47–10.57) and 5.01 (95% CI: 4.36–5.76) in the cannabis and the controls group. The aHR was 2.27 (95%CI: 1.66–3.11). No difference was observed between males and females (*P*-value for interaction > 0.05).

**Conclusions:**

Our study observed a short-term increased risk of ED visit/hospitalization for poisoning or for mental/behavioural disorders (from use of psychoactive drugs and other substances)- in medically authorized cannabis patients.

## Résumé

**Objectifs:**

L’intoxication par les médicaments psychoactifs et des troubles d’utilisation de substances (TUS) ont été rapportés chez les utilisateurs de cannabis non médical. Toutefois, on en sait peu sur les utilisateurs de cannabis médical et leur risque d’intoxication ou de développement d’un TUS. La présente étude a évalué le risque de visites au service d’urgence ou d’hospitalisation pour 1) une intoxication aux médicaments psychoactifs et 2) des troubles mentaux/comportementaux liés à l’utilisation de médicaments psychoactifs et d’autres substances psychoactives, chez des patients ayant reçu une autorisation médicale d'utilisation du cannabis en Ontario, Canada de 2014 à 2017.

**Méthodes:**

Étude de cohorte de patients adultes autorisés à utiliser du cannabis médical qui ont été appariés à des témoins témoins sélectionnés dans la population générale. Les visites au SU/hospitalisations ont été évaluées par un code diagnostique principal pour : 1) l’intoxication aux médicaments psychoactifs; 2) les troubles mentaux et comportementaux liés à l’utilisation de médicaments psychoactifs et d’autres substances psychoactives. Des modèles conditionnels de régression de Cox à risque proportionnel ont été conduits.

**Résultats:**

18 653 patients exposés au cannabis ont été appariés à 51 243 témoins. Durant un suivi médian de 243 jours, le taux d’incidence de l’intoxication était de 4,71 par 1000 années-personnes (IC à 95 % 3,71 à 5,99) pour les exposés et de 1,73 par 1000 années-personnes (IC à 95 % 1,36 à 2,19) pour les témoins. Le rapport de risques ajusté (RRa) était de 2,45 (IC à 95 % 1,56-3,84). Pour les troubles mentaux ou comportementaux, les taux d’incidence étaient de 8,89 (IC à 95 % 7,47 à 10,57) et de 5,01 (IC à 95% 4,36 à 5,76) dans le groupe du cannabis et des témoins, respectivement. Le RRa était de 2,27 (IC à 95 % 1,66 à 3,11). Aucune différence n’a été observée entre les hommes et les femmes (valeur-p d'interaction >0,05).

**Conclusions:**

Notre étude a observé un risque accru à court terme de visites au SU/hospitalisations pour intoxication ou troubles mentaux/comportementaux liés à l'utilisation des médicaments psychoactifs et d’autres substances psychoactives, chez des patients autorisés à utiliser du cannabis médicalement.

## Introduction

The use of non-medical cannabis has been reported to be highly correlated with the use of other psychoactive drugs including illegal, legal and prescribed drugs, as well as substance use disorders.^1–4^ Indeed, evidence suggests that non-medical cannabis use, particularly early onset use, is associated with greater risk of developing alcohol,^5^ and drug use disorders.^2–4^

However, evidence on the incidence of poisoning and substance use disorders amongst medical cannabis users is scarce. Patients usually seek cannabis for the treatment of pain, neurological and psychiatric disorders.^6,7^ Therefore, they may also use other drugs for the symptomatic treatment of these diseases such as opioids, anxiolytics, antidepressants and antiepileptic drugs^7,8^ and could potentially be at increased risk for poisoning or developing substance use disorders. Tetrahydrocannabinol (THC) and cannabidiol (CBD) are substrates and inhibitors of cytochromes enzymatic pathways implicated in metabolism of commonly prescribed agents,^8–10^ and therefore, the co-use of cannabis and these drugs could increase the risk of intoxication. For example, interactions between cannabinoids and antidepressants may be due to metabolizing enzyme inhibition which may, in some instances, lead to an increase of the antidepressants levels or their active metabolites resulting in an exacerbation of side effects such as serotonin syndrome.^8^

The small studies conducted among medical cannabis users showed contradictory results. Nugent et al. showed that medical cannabis users had higher risk for prescription opioid misuse, and higher rates of nicotine and alcohol use.^11^ However, in other cross-sectional surveys, patients reported seeking cannabis to substitute prescription agents for pain, anxiety, depression and sleep or illicit drugs, alcohol and other substances.^6,7,12,13^

In all, clarity is needed on medical cannabis use and the risk of psychoactive drugs and substance use disorders or intoxication. To address the evidence gap, this study assessed the risk of emergency department (ED) visit or hospitalization for 1) psychoactive drug-related poisoning, and 2) mental and behavioural disorders due to psychoactive drugs and other psychoactive substances among patients who were authorized to access medical cannabis in Ontario, Canada between 2014 and 2017.

## Materials (or Patients) and Methods

### Study Design

This retrospective longitudinal matched cohort study is part of a large study assessing the health outcomes of patients who received medical authorization to use cannabis for a wide range of health conditions in Ontario between April 24, 2014 and March 31, 2017. The date of cannabis authorization is the cannabis patient's index date. A description of an early update of the cannabis cohort is previously published.^14^ Ethics approval for this research was obtained from the University of Alberta Health Research Ethics Board (PRO 00083651) and Veritas Research Ethics Board (Ontario) (16111-13:21:103-01-2017).

To proceed to control matching, first, an index date is assigned to each patient who is eligible to be selected as control (from the general population) so that the distribution of the eligible controls index dates is similar to that of the cannabis patients. Next, baseline characteristics were assessed at the index date (i.e., sociodemographic variables), in the two years before the index date (i.e., healthcare utilizations) and in the 10 years before the index date for comorbidities. Finally, each cannabis patient was matched to up to three controls based on age (±1 years), sex, Local Health Integration Network location, income quartile, and history of health conditions including diabetes, heart disease, chronic obstructive pulmonary disease, asthma, cancer, musculoskeletal issues, neurological issues, pain, behavioral issues, fatigue, malnutrition, and other metabolic diseases. Matching was completed with replacement and thus an unauthorized patient could have been utilized for one or more authorized patients.

Each patient's follow-up was estimated from the index date and up to March 31st, 2017 within the administrative data.

### Study Population

The study population consisted of adult patients who were formally authorized (i.e., medical and administrative authorization) to access cannabis for medical use from a group of specialized cannabis clinics in Ontario (ON), Canada based. Patients could be referred to the clinics by their physicians or self-referred. Forms of authorized cannabis use included inhaled (smoked or vaporized) and orally consumed (oils). From these patients, we included individuals ≥ 18 years of age, of any sex and ethnicity, authorized for medical cannabis for a wide range of health conditions (acute and chronic), and those who were insured by the Ontario Health Insurance Plan (individuals had to be residents of ON). Patients from the general population with no record of a referral to a participating cannabis clinic were considered as controls.

We excluded patients who visited the cannabis clinics but did not receive an authorization from the controls pool as they may subsequently look for cannabis from other sources, those who received an authorization to use cannabis but were unable to be matched with at least one control, and those with invalid or duplicate identifiers. Finally, controls with cannabis-related ICD-10 codes (T407 and F12) were excluded.

### Data Source

This study is mainly based on the Ontario administrative health data for both cannabis patients and controls.

The Institute for Clinical Evaluative Sciences (ICES) provided the Ontario administrative data. These data include individual data files for each beneficiary, inpatient files, physician billings (inpatient and outpatient physician services) and prescription drug claims (84). The Ontario Health Insurance Plan (OHIP) contains information on physician services, including diagnostic codes. The Discharge Abstract Database (DAD) and the National Ambulatory Care Reporting System (NACRS) contain all data on hospitalizations and emergency department visits, respectively. For each emergency visit or hospitalization, up to 25 possible diagnoses were registered according to the *International Classification of Diseases* system- *tenth Revision* (*ICD-10*). Of these entries, only one indicated the most reliable diagnosis or the main diagnosis. The administrative databases were linked using the unique and encrypted patient health insurance number and covered the period of April 24, 2012 to March 31, 2017. We have previously assessed the healthcare utilizations of the cannabis cohort compared to controls using these data.^15^

### Outcomes

We assessed two main outcomes. The first outcome was defined as an ED visit or hospitalization with a main (primary) diagnosis code for poisoning by psychoactive drugs. This definition includes ICD-10 codes T40-poisoning by narcotics and hallucinogens (opium, heroin, cocaine, cannabis, lysergide and other), T41-posoining by anaesthetics and therapeutic gases (inhaled, intravenous, or local anesthetics, etc.), T42- poisoning by antiepileptic, sedative-hypnotic and antiparkinsonism drugs (barbiturates, benzodiazepines, iminostilbenes, etc) and T43-poisoning by psychoactive drugs, not elsewhere classified (tricyclic and tetracyclic antidepressants, monoamine-oxidase-inhibitor antidepressants, butyrophenone and thioxanthene neuroleptics, etc.). The complete description of the ICD-10 codes are provided Appendix in 1.

The second outcome was defined as an ED visit or hospitalization with a main diagnosis code for mental or behavioural disorders due to the use of psychoactive drugs and other psychoactive substances. This includes alcohol (ICD-10 F10), opioids (F11), cannabis (F12), Sedative, hypnotic or anxiolytic (F13), cocaine (F14), Other stimulant (F15), hallucinogen (F16), nicotine dependence (F17), inhalant (F18) and other psychoactive substances (F19) (See online supplemental material).

### Other Variables

Demographic variables included age at index date, sex, nearest census-based neighborhood income quintile and area of residence (rural vs. urban). We also considered patients’ existing health conditions including diabetes, congestive heart failure, chronic obstructive pulmonary disease, asthma, cancer, musculoskeletal issues, pain, neurologic disorders, fatigue, metabolic disease, mental health, and behavioral issues, liver disorders, and chronic kidney disease. Finally, we assessed specifically prior ED visits or hospitalization for 1) poisoning by psychoactive drugs, 2) mental and behavioural disorders due to psychoactive drugs, 3) mental and behavioural disorders due to alcohol use, and 4) other mental and behavioural disorders (See online supplemental material).

### Statistical Analysis

Descriptive statistics were used to assess the characteristics of the study sample (mean and standard deviation or median for continuous variables; numbers and proportions for categorical variables). Incidence rates for each outcome per 1,000 person-years were calculated for each group. Conditional Cox proportional hazards regressions, that account for the matching, were used to assess the association between cannabis use and the risk of ED visit or hospitalization for each outcome. The conditional models were further adjusted for variables not included in the matching. Analyses were first conducted to assess correlation between variables, the proportional hazards assumption, and to assess variables causing models’ misspecification. For example, for the first outcome (poisoning), including prior ED visit or hospitalization for mental and behavioural disorders due to psychoactive drugs in the model resulted in an extremely large hazard ratio (HR) for this variable. Thus, a composite variable that grouped prior ED visit or hospitalization for mental and behavioural disorders due to 1) psychoactive drugs, 2) alcohol and 3) other mental and behavioural disorders was created for this analysis. Hazard ratios and 95% confidence intervals (95%CI) were derived from each model.

In sensitivity analyses, we stratified each outcome-specific analysis by sex to assess possible sex-differences. For all analyses, a two-side *P *< 0.05 was considered as statistically significant. The analyses were performed using SAS version 9.4 (SAS Institute, Cary, NC, USA).

## Patient Consent

Informed consent was provided by the patient to the clinics at the time of first intake, which allows data to be collected and used for clinical and research purposes. The administrative data were provided by the Institute for Clinical Evaluative Sciences (ICES) administrative databases in Ontario and all data was released as de-identified data.

## Results

**Figure 1.**
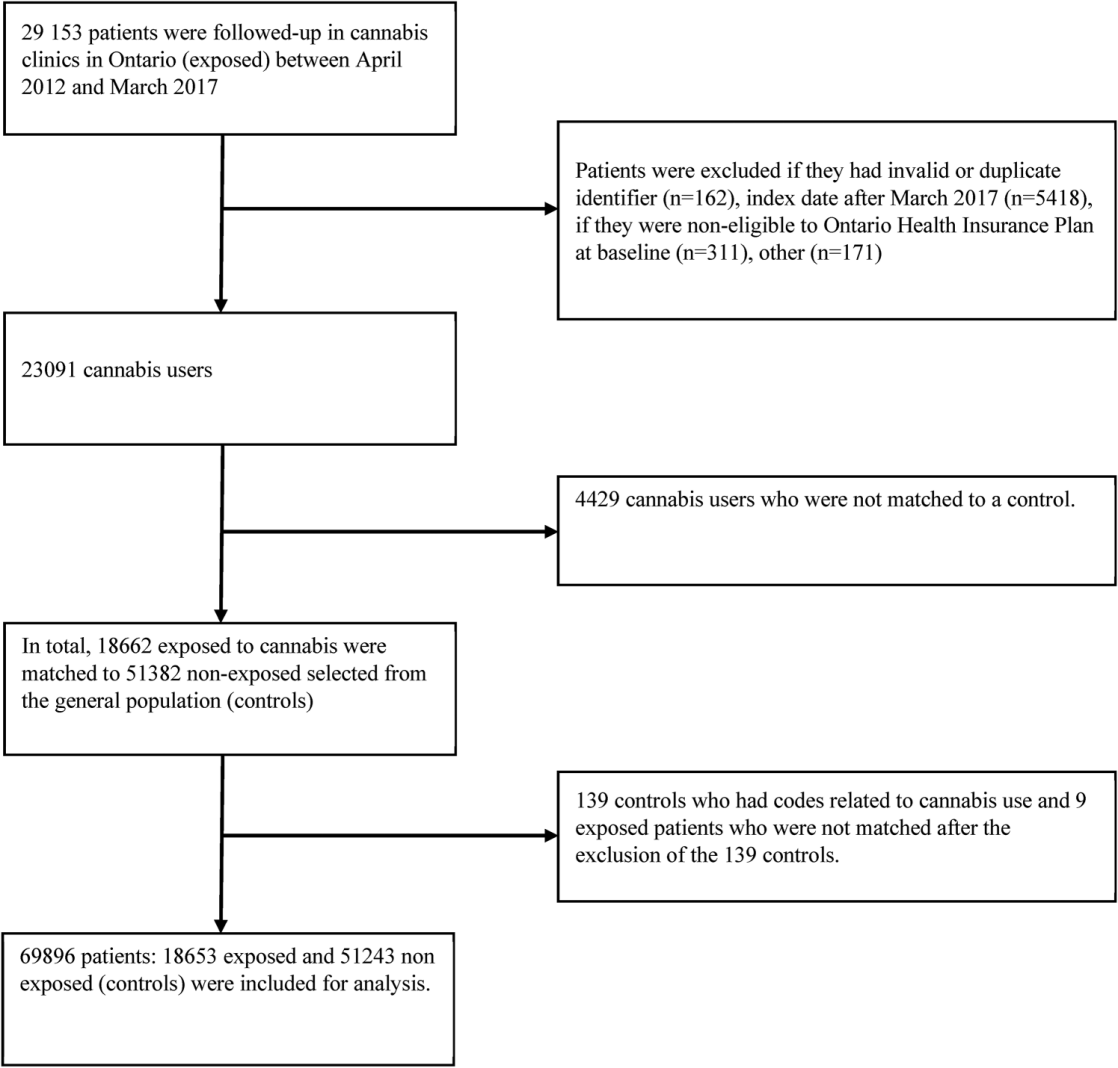
Selection of study population.

From 29,153 adult patients who received medical authorization to use cannabis, 18,653 were matched to 51,243 controls (Figure 1). The majority of exposed and non-exposed patients were aged 31 to 60 years and 54% were male. The most prevalent morbidities were respectively musculoskeletal disorders, asthma, behavioral disorders, neurological disorders and metabolic diseases (Table 1).

**Table 1.** Characteristics of the Study Sample.

Characteristics	General population (not authorized for medical cannabis) N = 51,243 (%)		Medically authorized cannabis patients N = 18,653 (%)	
Age, years				
<21	331	(0.65)	119	(0.64)
21–30	5,578	(10.89)	1,972	(10.57)
31–40	10,088	(19.69)	3,604	(19.32)
41–50	10,545	(20.58)	3,822	(20.49)
51–60	13,227	(25.81)	4,842	(25.96)
61–70	7,771	(15.16)	2,858	(15.32)
71–80	2,745	(5.36)	1,050	(5.63)
>81	958	(1.87)	386	(2.07)
Sex				
Female	23,206	(45.29)	8,528	(45.72)
Male	28,037	(54.71)	10,125	(54.28)
Nearest census based neighbourhood income quintile				
1	10,943	(21.36)	4,053	(21.73)
2	10,524	(20.54)	3,859	(20.69)
3	9,943	(19.40)	3,595	(19.27)
4	10,327	(20.15)	3,726	(19.98)
5	9,506	(18.55)	3,420	(18.33)
Rural	6,046	(11.80)	1,798	(9.64)
Morbidities considered in initial matching (based on administrative data)				
Asthma	9,478	(18.50)	3,690	(19.78)
Behavioural disorders	8,800	(17.17)	3,573	(19.16)
Cancer	4,472	(8.73)	1,828	(9.80)
Congestive heart failure	295	(0.58)	166	(0.89)
Chronic obstructive pulmonary disease	5,722	(11.17)	2,351	(12.60)
Diabetes	5,390	(10.52)	2,214	(11.87)
Fatigue	460	(0.90)	277	(1.49)
Metabolic disease	5,945	(11.60)	2,605	(13.97)
Musculoskeletal disorders	21,716	(42.38)	8,250	(44.23)
Neurological disorders	6,812	(13.29)	2,886	(15.47)
Liver disorders	705	(1.38)	475	2.55
Chronic kidney disease	518	(1.01)	281	(1.51)
Prior ED visit or hospitalization for				
Poisoning by psychoactive drugs	140	(0.27)	133	(0.71)
Mental and behavioural disorders due to psychoactive drugs	224	(0.44)	152	(0.81)
Mental and behavioural disorders due to alcohol use	352	(0.69)	106	(0.57)
Other mental and behavioural disorders	1,520	(2.97)	842	(4.51)
Baseline health conditions for patients with cannabis authorization (most likely reasons for seeking cannabis, based on initial clinical assessment)*	NA	NA		
Any pain (includes cancer pain)			14,297	81.08
Shoulder pain			2,265	12.85
Back pain			7,365	41.77
Neck pain			2,329	13.21
Leg pain			2,679	15.19
Knee pain			2,123	12.04
Hip pain			1,784	10.12
Fibromyalgia			1,880	10.66
Neurologic pain			1,708	9.69
Migraine/headache			1,839	10.43
Multiple sclerosis			3,274	18.57
Any mental health			7,800	44.24
Anxiety			4,850	27.51
Depression			3,389	19.22
Post-traumatic stress disorders			846	4.80
Sleep problems			3,948	22.39

* 1,020 missing values (5.4%); most prevalent conditions are reported.

During a median follow-up of 243 days (Q1 = 113; Q3 = 402), the incidence rate for poisoning by psychoactive drugs was 4.71 per 1,000 person-years (95%CI: 3.71–5.99) for authorized medical cannabis patients and 1.73 per 1,000 person-years (95% CI: 1.36–2.19) in the control group (Table 2). Similar rates were observed among males and females in both groups (Table 3).

**Table 2.** Incidence Rates of Emergency Department Visit or Hospitalization for 1) Poisoning by Psychoactive Drugs and 2) Mental and Behavioural Disorders due to use of Psychoactive Drugs and Other Psychoactive Substances.

Outcome	Group	Number of events	Total person-years	Incidence rates per 1,000 person-years (95% CI)
Poisoning by psychoactive drugs	Cannabis cohort	67	14,208.39	4.71 (3.71–5.99)
	Controls	68	39,419.54	1.73 (1.36–2.19)

Mental and behavioural disorders due to use of psychoactive drugs and other substances	Cannabis users	126	14,171.83	8.89 (7.47–10.58)
	Controls	197	39,334.42	5.01 (4.36–5.76)

**Table 3.** Sex-Specific Incidence Rates of Emergency Department Visit or Hospitalization for 1) Poisoning by Psychoactive Drugs and 2) Mental and Behavioural Disorders due to use of Psychoactive Drugs and Other Psychoactive Substances.

Group	Sex	Number of events	Total person-years	Incidence rates per 1,000 person-years (95% CI)
**Poisoning by psychoactive drugs**				
Cannabis cohort	Males	37	7,920.61	4.61 (3.39–6.44)
	Females	30	6,287.77	4.77 (3.34–6.82)
Controls	Males	39	21,544.17	1.81 (1.32–2.48)
	Females	29	17,875.37	1.62 (1.13–2.33)
**Mental and behavioural disorders due to use of psychoactive drugs and other substances**				
Cannabis users	Males	90	7,881.72	11.42 (9.30–14.02)
	Females	36	6,290.11	5.72 (4.13–7.93)
Controls	Males	143	21,473.96	6.66 (5.66–7.84)
	Females	54	17,860.47	3.02 (2.32–3.95)

In the total sample, the adjusted hazard ratio (aHR) for poisoning by psychoactive drugs was 2.45 (95%CI: 1.56–3.84) (Table 4). Although the aHR among females was higher than among males, there was no difference in the risk between males and females (the interaction between sex and cannabis authorization was non-significant, *P*-value > 0.05).

**Table 4.** Association Between the Medical cannabis Authorization and Risk of Emergency Department Visit or Hospitalization for 1) Poisoning by Psychoactive Drugs and 2) Mental and Behavioural Disorders due to the use of Psychoactive Drugs and Other Psychoactive Substances.

	Adjusted hazard ratio1 (95%CI)*	Adjusted hazard ratio2 (95%CI)**
Outcome 1 = ED visit or hospitalization for poisoning by psychoactive drugs		
Total sample	2.52 (1.63–3.90)	2.45 (1.56–3.84)^≠^
Males	2.12 (1.22–3.69)	1.87 (1.04–3.38)^¥^
Females	3.33 (1.63–6.80)	3.52 (1.68–7.39)^£^
Outcome 2 = ED visit or hospitalization for mental and behavioural disorders due to psychoactive drugs and other substances		
Total sample	1.66 (1.27–2.15)	2.27 (1.66–3.11)π
Males	1.54 (1.13–2.09)	2.13 (1.47–3.09)π
Females	2.03 (1.21–3.39)	2.83 (1.53–5.23)^π^

* Conditional Cox regression model (accounts for all matching variables: age, sex, income quartile and previous diagnosis of diabetes, congestive heart failure, chronic obstructive pulmonary disease, asthma, cancer, musculoskeletal disorders, neurological disorders, pain, fatigue, behavioural disorders, malnutrition, and metabolic disease).

** The conditional model was further adjusted for variables mentioned below (difference in variables considered in each model was to avoid models’ misspecification with certain variables).

^≠^ Adjusted for area of living (rural versus urban), liver disorders and chronic kidney disease, and ED visit or hospitalization for 1) poisoning by psychoactive drugs and 2) any mental and behavioural disorders.

^¥^ Adjusted for liver disorders and ED visit or hospitalization for 1) poisoning by psychoactive drugs and 2) any mental disorders.

^£^ Adjusted for area of living (rural versus urban), ED visit or hospitalization for 1) poisoning by psychoactive drugs and 2) any mental and behavioural disorders.

^π^ Adjusted for area of living (rural versus urban), liver disorders, and ED visit or hospitalization for 1) poisoning by psychoactive drugs, 2) mental and behavioural disorders due to psychoactive drugs, 3) alcohol-related mental and behavioural disorders and 4) other mental and behavioural disorders.

For mental and behavioural disorders due to psychoactive drugs or substance use, the incident rates were 8.89 (95% CI: 7.47–10.57) and 5.01 (95% CI: 4.36–5.76) in the cannabis group and the controls group, respectively (Table 2). The aHR for this outcome was 2.27 (95%CI: 1.66–3.11) (Table 4). No difference was observed between males and females (*P*-value for interaction > 0.05).

## Discussion

From 2014–2017, this cohort study of Ontario adult patients showed that medical cannabis authorization observed a short-term increased risk of ED visit or hospitalization for poisoning by psychoactive drugs and for mental and behavioural disorders due to use of psychoactive drug and other psychoactive substances. The risk was similar among males and females. As mental health conditions are among the primary reasons for seeking medical cannabis,^7^ these findings suggest that frontline clinicians should consider existing risk factors including previous drug poisoning and mental health disorders, prior to cannabis authorization.

The observation that medical cannabis users are at higher risk of poisoning by psychoactive drugs is biologically plausible. Indeed, the two main components of cannabis plant, Δ9 -Tetrahydrocannabinol and cannabidiol, are inhibitors of cytochrome P450 enzymatic pathways relevant to the biotransformation of commonly prescribed psychoactive agents.^9^ Thus, this inhibition of the cytochrome P450 may potentially increase the risk of intoxication by psychoactive drugs that are used simultaneously with cannabis. It is also suggested that heavy and frequent use of cannabis may increase vulnerability to mental health disorders including substance use disorder, through an alteration of the anticipatory reward processing over time.^16,17^

Our results are consistent with those of one of the rare clinical studies that assessed problematic cannabis use among specifically medical cannabis patients. In this prospective study that included 265 medical cannabis patients (without a control group), 26% and 9% had cannabis misuse and addiction behavior, respectively, during a 12-month follow-up.^18^ Nugent et al. also observed higher rates of prescription opioid misuse, and hazardous alcohol use among medical cannabis users compared to non-users of cannabis, both groups being chronic opioid users at baseline.^11^ In a US nationwide prospective study that was not specific to medical cannabis use, Blanco et al. also showed that cannabis use predicted an increased incidence of other substance use disorders.^4^ However, the results contrast with patients’ survey data which suggest that patients are seeking cannabis to substitute opioids, alcohol and other psychoactive substances.^6,7,12,13^

Despite the possible sex differences in the endocannabinoid system that could differentially affect cannabis response in males and females,^19^ no significant difference in the risk of poisoning by psychoactive drugs or mental and behavioural disorders due to the use of psychoactive drugs and other substances was observed between males and females in our analysis. However, the observed higher estimate of mental and behavioural disorders among females needs to be further investigated and may also suggest that females could be potentially at higher risk.

The main strengths of this study are the large number of medical cannabis users included (probably the largest medical cannabis cohort), the possibility to match these cannabis patients with population-based controls, and the assessment of outcomes that reflect a high degree of seriousness (i.e. requiring ED visit or hospitalization) and less subject to self-reporting bias. Our study provides new data on drugs and substances-related use disorders among specifically the medical cannabis users, an understudied research question.

Among the limitations, about 19% of the cannabis patients were excluded from the analysis as it was not possible to match them to at least one control. This issue has probably led to an underestimation of the study outcomes in the cannabis group as the excluded patients were more likely to be older and had higher rates of morbidities. As this is an observational study, there is potential for spectrum bias as our cohort of patients were adults who individually sought medical cannabis. Consequently, we are unable to make a causal conclusion regarding medical cannabis use and hospitalization due to poisoning. A second limitation is the non-adjustment of the analysis for prescribed psychoactive drugs as data on drugs were only available for a subset of the sample. However, by adjusting for previous drug-related mental disorders and poisoning, we were able to potentially minimize this bias. Thirdly, we were unable to account for the specific cannabinoids used by the patients and their modes of consumption that could potentially affect the assessed risks differentially. Indeed, there is uncertainty as to whether the medical cannabis authorized was consumed as prescribed and whether the patients elected to consume or use other treatments. Lastly, given wide variability of the type of cannabis products or cannabis cultivars used, we cannot pinpoint one specific strain or dose of medical cannabis that may have attributed to hospitalizations due to poisoning.

## Conclusion

In all, our study observed a short-term increased risk of ED visits and hospitalization for poisoning by psychoactive drugs as well as mental and behavioural disorders in our medically authorized cannabis cohort– as a result of psychoactive drugs and other substance use. No significant difference in the risk was observed between males and females. Our study findings provide ongoing evidence and information to frontline clinicians regarding potential hospitalization risks for drugs use disorders for current and prospective medical cannabis users.

## Supplemental Material

sj-docx-1-cpa-10.1177_07067437211060597 - Supplemental material for Substance Use Disorders and Psychoactive Drug Poisoning in Medically Authorized Cannabis Patients: Longitudinal Cohort Study Supplemental material, sj-docx-1-cpa-10.1177_07067437211060597 for Substance Use Disorders and Psychoactive Drug Poisoning in Medically Authorized Cannabis Patients: Longitudinal Cohort Study by Arsène Zongo, Cerina Lee, Jihane El-Mourad, Jason R. B. Dyck, Elaine Hyshka, John G. Hanlon and Dean T. Eurich in The Canadian Journal of Psychiatry
