# Supplementary material for: Substance Use Disorders and Psychoactive Drug Poisoning in Medically Authorized Cannabis Patients: Longitudinal Cohort Study
Source: Can J Psychiatry. 2021 Nov 20;67(7):544–52. doi: 10.1177/07067437211060597 (PMC9234898; doi:10.1177/07067437211060597)
Supplement: sj-docx-1-cpa-10.1177_07067437211060597 - Supplemental material for Substance Use Disorders and Psychoactive Drug Poisoning in Medically Authorized Cannabis Patients: Longitudinal Cohort Study [file sj-docx-1-cpa-10.1177_07067437211060597.docx]

**Appendix A: Definitions of the Study Outcomes and variables**

| **Outcomes** | **ICD-10 Codes** |
| --- | --- |
| ED visit or hospitalization with  a main diagnosis code  (primary diagnosis code)  for poisoning by psychoactive drugs | **T40 – Poisoning by narcotics and psychodysleptics (hallucinogens)**  T40.0 Poisoning by opium  T40.1 Poisoning by heroin  T40.2 Poisoning by other opioids (Includes: Codeine, Morphine)  T40.3 Poisoning by methadone  T40.4 Poisoning by other synthetic narcotics (Includes: Pethidine)  T40.5 Poisoning by cocaine  T40.6 Poisoning by other and unspecified narcotics  T40.7 Poisoning by cannabis (derivatives)  T40.8 Poisoning by lysergide (LSD)  T40.9 Poisoning by other and unspecified psychodysleptics (hallucinogens) (Includes: Mescaline, Psilocin, Psilocybine)  **T41- Poisoning by anaesthetics and therapeutic gases**  T41.0 Poisoning by inhaled anaesthetics  Excludes: oxygen (T41.5)  T41.1 Poisoning by intravenous anaesthetics  Includes: Thiobarbiturates  T41.2 Poisoning by other and unspecified general anaesthetics  T41.3 Poisoning by local anaesthetics  T41.4 Poisoning by anaesthetic, unspecified  T41.5 Poisoning by therapeutic gases (Includes: Carbon dioxide, Oxygen)  **T42- Poisoning by antiepileptic, sedative-hypnotic and antiparkinsonism drugs**  T42.0 Poisoning by hydantoin derivatives  T42.1 Poisoning by iminostilbenes  Includes: Carbamazepine  T42.2 Poisoning by succinimides and oxazolidinediones  T42.3 Poisoning by barbiturates  Excludes: thiobarbiturates (T41.1)  T42.4 Poisoning by benzodiazepines  T42.5 Poisoning by mixed antiepileptics, not elsewhere classified  T42.6 Poisoning by other antiepileptic and sedative-hypnotic drugs (Includes: Methaqualone, Valproic acid)  T42.7 Poisoning by antiepileptic and sedative-hypnotic drugs, unspecified  T42.8 Poisoning by antiparkinsonism drugs and other central muscle-tone depressants (Includes: Amantadine)  **T43 – Poisoning by psychoactive drugs, not elsewhere classified**  T43.0 Poisoning by tricyclic and tetracyclic antidepressants  T43.1 Poisoning by monoamine-oxidase-inhibitor antidepressants  T43.2 Poisoning by other and unspecified antidepressants  T43.3 Poisoning by phenothiazine antipsychotics and neuroleptics  T43.4 Poisoning by butyrophenone and thioxanthene neuroleptics  T43.5 Poisoning by other and unspecified antipsychotics and neuroleptics  T43.6 Poisoning by psychostimulants with abuse potential  T43.8 Poisoning by other psychoactive drugs, not elsewhere classified  T43.9 Poisoning by psychoactive drug, unspecified |
| ED visit or hospitalization  with a main diagnosis  (primary diagnosis code)  for mental or behavioral disorders  due to the use of psychoactive drugs  or other substances | F10 – Alcohol related disorders  F11 – Opioid related disorders  F12 – Cannabis related disorders  F13 – Sedative, hypnotic or anxiolytic related disorders  F14 – Cocaine related disorders  F15 – Other stimulant related disorders  F16 – Hallucinogen related disorders  F17 – Nicotine dependence  F18 – Inhalant related disorders  F19 – Other psychoactive substance related disorders |
| **Covariates** |  |
| Prior ED visit or hospitalization for  poisoning by psychoactive drugs | T40, T41, T42, T43 |
| Prior ED visit or hospitalization for mental  and behavioural disorders due to  psychoactive drugs | F11, F12, F13, F14, F15, F16, F18, F19 |
| Prior ED visit or hospitalization for mental  and behavioural disorders due to alcohol use | F10 |
| Prior ED visit or hospitalization for other mental and behavioural disorders | F00 to F99, excluding  F11, F12, F13, F14, F15, F16, F18, F19 and F10 |
